# Telomere biology and its maintenance in schizophrenia spectrum disorders: Exploring links to cognition

**DOI:** 10.1016/j.schres.2024.08.011

**Published:** 2024-10

**Authors:** Vid Mlakar, Ibrahim Akkouh, Els F. Halff, Deepak P. Srivastava, Viktoria Birkenæs, Torill Ueland, Daniel S. Quintana, Monica B.E.G. Ormerod, Nils Eiel Steen, Srdjan Djurovic, Ole A. Andreassen, Monica Aas

**Affiliations:** a Social, Genetic and Developmental Psychiatry Centre, Institute of Psychiatry, Psychology and Neuroscience, King's College London, London, UK; b Department of Medical Genetics, Oslo University Hospital, Oslo, Norway; c Department of Basic and Clinical Neuroscience, Institute of Psychiatry, Psychology and Neuroscience, King's College London, London, UK; d Centre for Precision Psychiatry, Division of Mental Health and Addiction, University of Oslo, Norway; e Section for Clinical Psychosis Research, Division of Mental Health and Addiction, Oslo University Hospital, Norway; f Department of Psychology, University of Oslo, Oslo, Norway; g NevSom, Department of Rare Disorders, Oslo University Hospital, Oslo, Norway; h Division of Mental Health and Substance Abuse, Diakonhjemmet Hospital, Oslo, Norway; i Department of Clinical Science, University of Bergen, Bergen, Norway; j Department of Psychosis Studies, Institute of Psychiatry, Psychology & Neuroscience, King's College London, London, England, UK.; k MRC Centre for Neurodevelopmental Disorders, King's College London, London, UK

**Keywords:** Telomeres/telomere length maintenance and repair, Telomerase expression, Cognitive impairments, Schizophrenia spectrum disorders

## Abstract

**Objective:**

Contemporary research suggests reduced telomere length in schizophrenia spectrum disorders (SZ) compared to age-adjusted non-affected individuals. However, the role of telomere maintenance and telomere repair in SZ is poorly understood as well as the involvement of telomere biology in cognitive abnormalities in SZ.

**Methods:**

The study consisted of 758 participants (SZ [*n* = 357] and healthy controls, HC [*n* = 401]) collected as part of the Norwegian TOP study. Participants were assessed with standardized neuropsychological tests measuring five cognitive domains. Leucocyte telomere length (TL) was measured via blood and determined by quantitative real-time Polymerase Chain Reaction (qPCR) providing a telomere to single copy ratio (T/S ratio), used to estimate the mean telomere length. Telomerase activity was assessed by the expression levels of the Telomerase Reverse Transcriptase (*TERT*) and Telomerase RNA Component (*TERC*) genes. To assess telomere maintenance and telomere repair we calculated the telomerase expression to TL ratio (*TERT*/TL and *TERC*/TL respectively).

**Results:**

Patients had reduced *TERT* (*F* = 5.03, *p* = 0.03), but not *TERC* expression (*F* = 1.04, *p* = 0.31), and higher *TERT*/TL (*F* = 6.68, *p* = 0.01) and *TERC*/TL (*F* = 6.71, *p* = 0.01), adjusted for age, sex, and ethnicity. No statistically significant association was observed between any of the telomere biology markers and the cognitive domains (*p* > 0.05).

**Conclusion:**

Our study shows changes in *TERT* expression and telomere maintenance and telomere repair in SZ compared HC. However, the role of telomere biology in the mechanism underlying cognitive impairment in psychosis seems limited.

## 1 Introduction

Individuals affected by schizophrenia spectrum disorders (SZ) have significantly shorter life expectancy than to the general population (Dregan et al., 2020; Correll et al., 2022; Chang et al., 2023). This is concurrent with an increased risk of developing somatic diseases associated with advanced age, such as cardiovascular diseases, metabolic syndrome, immune dysregulation, and dementia (Rødevand et al., 2023; Launders et al., 2022; Kruckow et al., 2023). Older age is associated with poorer cognitive performance, especially in verbal learning and memory (Harada et al., 2013; Lupien et al., 2007; Powell et al., 2018), domains which are also frequently impaired in SZ (Aas et al., 2014). Cognitive impairment is considered a core feature of SZ (Aas et al., 2014) and individuals with the disorder on average perform one standard deviation below that of healthy individuals across cognitive domains (McCutcheon et al., 2023). However, the underlying pathophysiology of cognitive impairment is currently unclear. Emerging research indicates that shorter life expectancy and cognitive impairments could be linked to an accelerated rate of biological ageing as measured by shorter telomere length and reduced telomerase activity within this population (Teeuw et al., 2021; Lin et al., 2021; Constantinides et al., 2023, Gurvich et al., 2023).

Telomeres are repeating TTAGGG nucleotide structures located on the ends of all human chromosomes protecting them against progressive DNA deteriorating (Blackburn, 2005). Whilst telomeric degeneration is a universal process, epidemiological studies indicate variations in telomere length associated with biological sex (Lansdorp, 2022; Ng and Hazrati, 2022; Olivieri et al., 2023), ancestry (Needham et al., 2019), severe mental disorders (Ayora et al., 2022; Mlakar et al., 2023; Mutz and Lewis, 2022) and disorders of cognitive decline, such as Alzheimer's and Parkinson's disease (Rodríguez-Fernández et al., 2022; Kuan et al., 2023; Liu et al., 2023). Evidence also suggests variation in key telomere maintenance mechanisms, namely telomerase activity and the expression of telomerase reverse transcriptase (*TERT)* and telomerase RNA component (*TERC)* genes, in both Alzheimer's (Yashooa and Nabi, 2022; Yu et al., 2023), Parkinson's (Wan et al., 2021; Vellingiri et al., 2023) and severe mental disorders (Lundberg et al., 2020; Bürhan-Çavuşoğlu et al., 2021; Walia et al., 2023).

To date, studies of telomere length in SZ have provided somewhat conflicting results. While most studies suggest that patients with SZ have shorter telomeres compared to healthy controls (HC) (Russo et al., 2018; Aas et al., 2019; Gurvich et al., 2023), others have found no significant differences (Riley et al., 2018; Omidpanah et al., 2019; Schürhoff et al., 2021). Finally, some studies have even reported longer telomeres in SZ patients compared to HC (Cui et al., 2017; Maurya et al., 2018; Zhang et al., 2018). Telomerase expression is less researched in SZ, with only one study indicating nominally decreased telomerase activity in SZ (Porton et al., 2008). Moreover, single nucleotide polymorphisms (SNPs) in the *TERT* gene have been associated with increased risk for developing SZ (Rao et al., 2016). Thus, investigating several markers of cellular ageing would be beneficial to increase our knowledge on the role of accelerated ageing and cognitive functioning in SZ.

Despite the existence of both potential accelerated ageing processes and cognitive deficits in SZ, less than a handful of studies have investigated how cognitive abnormalities may be related to telomere length in SZ. A study by Czepielewski et al. (2018) found that shorter telomeres in individuals with SZ were associated with reduced total grey matter volume, which is important for memory performance (Czepielewski et al., 2022). Another study by Gurvich et al. (2023) demonstrated an association between shorter telomere lengths and cognitive impairment in individuals with SZ applying a general cognitive measure without specifying the cognitive domain (Gurvich et al., 2023). However, both studies consisted of <50 individuals with SZ. The low sample sizes and number of studies highlight the need for larger, well-charactered samples investigating the role of telomere length and cognitive functioning in SZ. Studies investigating telomerase expression and cognitive function in this population are to date lacking.

The current study investigates the association between ageing processes (indexed by telomere length, expression levels of the telomerase *TERT* and *TERC* genes, and telomerase expression to telomere length ratios for both genes) and cognitive functioning in the largest sample to date of individuals with SZ and HC. We have previously shown that patients with a psychotic or bipolar disorder have shorter telomere length than HC (Aas et al., 2019), however this study is, to our knowledge, the first to investigate the role of telomerase maintenance and repair in psychotic disorders including measurements of telomerase expression and Telomerase/telomere length ratio. We expect to find reduced telomerase expression in SZ compared to HC. We hypothesize that cognitive impairment will be associated with shorter telomere lengths and reduced telomerase expression in SZ, and that this effect will be most prominent in cognitive domains known to be sensitive to ageing, such as memory and learning.

## 2 Methods

### 2.1 Participants

The study sample consisted of 758 participants (schizophrenia spectrum [SZ; *n* = 357] and healthy controls [HC; *n* = 401]), collected as part of the cross-sectional Thematically Organized Psychosis (TOP) study between 2007 and 2018 (Simonsen et al., 2011). The patients were sampled from psychiatric hospitals located throughout Oslo, Norway. Fifty-five percent of the patients had a diagnosis of schizophrenia, 9 % a schizophreniform, 15 % a schizoaffective, and 21 % with an “other psychosis” diagnosis. Healthy controls were recruited via random invitation, utilizing population registers of two counties in the Oslo metropolitan area (Oslo & Akershus). The inclusion criteria for HC were as follows: No current or lifetime diagnosis of a severe mental disorder or substance abuse or dependency and no severe mental disorders in their close relatives. The following exclusion criteria were imposed on the whole sample, namely: Age outside the 18–65 range, not being fluent in Norwegian and a current or past organic psychosis, neurological disorder or other medical condition which could impact cognitive functioning (Simonsen et al., 2011). The study was approved by the Regional Committee for Medical Research Ethics and the Norwegian Data Inspectorate, with all participants providing informed consent.

### 2.2 Clinical assessment

Clinical diagnoses of SZ patients were assessed by trained physicians, psychiatrists, and clinical psychologists, using the Structured Clinical Interview for DSM-IV (Axis I disorders (SCID-I), chapters A-E; Spitzer et al., 1992) and the Positive and Negative Syndrome Scale (PANSS) (Kay et al., 1987). Training was based on the SCID 101 training program from University College Los Angeles (UCLA, Ventura et al., 1998). The overall inter-rater reliability was found to be satisfactory with an overall kappa score between 0.92 and 0.99 across assessment teams (Høegh et al., 2020). Additional clinical information was collected during assessments, namely duration of illness (defined as current age minus age at onset of a first episode), current severity of symptoms and functioning (using the split Global Assessment of Functioning, GAF, Pedersen et al., 2007), and medication. Reviews of patient's medical charts facilitated collection of data on daily defined dose (DDD) and type of medication.

### 2.3 Cognitive assessment

All participants underwent neuropsychological testing, carried out by trained clinical psychologists (patients) or assistants (healthy controls). Participants included prior to 2012 were tested using the cognitive test battery described by Simonsen et al. (Simonsen et al., 2010; Battery 1), whilst participants enrolled after 2012 were examined using tests included in the MATRICS Consensus Cognitive Battery with the addition of delayed recall from the Hopkins Verbal Learning Test-Revised (Brandt and Benedict, 2001) (Battery 2). Supplementary Table S1 illustrates the five established cognitive domains and the assessments used by each corresponding battery respectively as previously published. To combine results from the two batteries, tests measuring the same cognitive domains (i.e., processing speed, working memory, verbal fluency, verbal memory, and verbal learning) were transformed into z-scores and merged as previously described and validated in Laskemoen et al., 2020. We have previously shown that patients have reduced cognitive function compared to controls (see for example Simonsen et al., 2011), however this is the first-time including cellular measures of accelerated ageing (telomere lengths and telomerase expression) within this context. All analyses are new, if not otherwise stated.

### 2.4 Telomere length

Telomere length was measured in peripheral blood leukocytes of all participants, utilizing a quantitative real-time polymerase chain reaction (qPCR) method (Aas et al., 2019; Mlakar et al., 2023). Firstly, 10 ng of DNA was extracted from peripheral blood leukocyte cells and combined with 5 μl of SYBR®Green JumpStart Taq Ready Mix and 0.25 μl of ROX reference dye. The primers for the telomeric reaction included: 300 nM TelA (5′-CGG TTT GTT TGG GTT TGG GTT TGG GTT TGG GTT TGG GTT-3′) and 900 nM TelB (5′-GGC TTG CCT TAC CCT TAC CCT TAC CCT TAC CCT TAC CCT-3′). Moreover, the single copy gene 36B4 included primers 200 nM 36B4F (5′- CAG CAA GTG GGA AGG TGT AAT CC 3′) and 400 nM 36B4R (5′-CCC ATT CTA TCA ACG GGT ACA A-3′). All telomere length measurements were assessed on a 384-well plate Applied Biosystems 7900HT Fast Real Time qPCR. Three DNA samples with pre-measured telomere lengths (10.4 kb, 3.9 kb and 2 kb) were ran as controls. Inconclusive samples, as well as samples in both the top and bottom 5 % were re-evaluated. The qPCR provided a telomere to single copy ratio (T/S ratio), which was used to estimate mean telomere length, with smaller T/S ratios indicating shorter mean telomere length. All blood samples were stored in The Biobank - Oslo, Norway. Analyses of variation coefficients revealed that the study sample possessed an intra-assay coefficient of 6.07 % and an inter-assay coefficient of 6.08 %.

### 2.5 *TERT* and *TERC* expression

Expression of *TERT* and *TERC* was measured using microarrays as previously described (Akkouh et al., 2018). Briefly, blood samples were collected in Tempus Blood RNA Tubes, and total RNA was extracted with the TEMPUS 12-Port RNA Isolation Kit (Thermo Fisher) according to manufacturer's protocol. Gene expression quantification was performed with Illumina HumanHT-12 v4 Expression BeadChip (Illumina). Multidimensional scaling and hierarchical clustering were used for regular quality control, including sample quality measurements and removal of outliers, as well as removal of multiple batch effects (RNA extraction batch, RNA extraction method, DNase treatment batch, cRNA labelling batch, and chip hybridization). Batch-adjusted and log2-transformed expression values of *TERT* and *TERC* were used for statistical analyses. *TERT/*TL and *TERC/*TL ratios were calculated to obtain a measure of telomere maintenance efficiency.

### 2.6 Statistical analysis

Statistical analyses were performed using the IBM SPSS v26 software. Chi-Squared tests were used for categorical variables (sex and ethnicity) to compare distribution between the two groups (patients with SZ and HCs).

ANCOVAs were performed to compare telomere length, telomerase (*TERT* and *TERC)* gene expression levels, and cognitive domains (working memory, verbal fluency, processing speed, verbal memory, and verbal learning), between SZ and HCs groups adjusted for age, sex, and ethnicity.

Linear regressions were used to analyse the relationship between telomere length/telomerase expression and cognition with each cognitive domain, analyzed one at the time as the dependent variable and telomere length or telomerase activity (*TERT*, *TERC*, *TERT/TL* ratio, and *TERC/TL* ratio) as the independent variable. Analyses were run in HC and SZ separately as well as testing for group interaction. For SZ, the analyses were adjusted for age, sex, ethnicity, and daily defined dose of antipsychotics, mood stabilizers, and antidepressants. For HC, all analyses were adjusted for age sex and ethnicity only. Due to the cognitive data collection being carried out in two waves, data from each test battery were also analyzed separately.

The main analyses (the role of telomere length/telomerase expression and cognitive functioning) were adjusted using a False Discovery Rate (FDR) correction (cellular ageing [telomere length, *TERT*, *TERC*, *TERT*/TL ratio, and *TERC*/TL ratio] and cognitive domains [working memory, verbal fluency, processing speed, verbal memory, and verbal learning] in patients and controls separately, and the group status [patient/control] interaction). As we anticipated shorter telomere length and reduced telomerase expression to be related to having poorer verbal memory and learning correlates in SZ, alpha level was set at 0.05 with FDR corrections.

## 3 Results

### 3.1 Demographic overview

Table 1 provides the demographic description of the sample (*n* = 758). The SZ group was significantly younger than the HC control group, and there were significantly more individuals with European ancestry in the HC group compared to the SZ group. However, there was no significant difference in sex between the groups (*χ*^*2*^ = 0.38, *p* > 0.05). In terms of medication, 2.2 % of the SZ sample were taking lithium, 83.5 % taking antipsychotic medication and 24.6 % taking antidepressant medication. Data regarding duration of illness and the GAF symptom and functioning scales for the SZ group are also reported in Table 1. As published previously from a subsample of the same cohort (Simonsen et al., 2011), patients with SZ performed poorer than HC on all cognitive domains (Table S2).

**Table 1:** Sample demographics.

Variable	SZ (*n* = 357)	HC (*n* = 401)	Statistics	Post hoc analyses
Age (years), mean (SD)	29.38 (9.36)	31.39 (7.63)	*F* = 6.34, *p* = 0.002	SZ < HC
Sex Male, N (%)	210 (58.8)	227(56.6)	*χ*^*2*^ = 0.38, *p* > 0.05	–
Ethnicity European, N (%)	284 (79.6)	393 (98)	*χ*^*2*^ = 69.78, *p* < 0.001	SZ < HC
Years of Education, mean (SD)	13.14 (2.87)			–
Duration of Illness, median (min-max)	4.00 (0–44)	–	*–*	–
Psychopathology (GAF)				
Symptom, median (min-max) Function, median (min-max)	40.00 (8–91) 42.00 (6–85)	–	–	–
Mood Stabiliser Dose (mg/day), mean (SD)	0.08 (0.27)	–	*–*	–
Antidepressant Dose (mg/day), mean (SD)	0.39 (0.83)	–	*–*	–
Lithium Dose (mg/day), mean (SD)	0.02 (0.12)	–	*–*	–
Antipsychotic Dose (mg/day), mean (SD)	1.91 (4.79.2)	–	*–*	–
Telomere Length (T/S ratio), mean (SE)	0.09 (0.03)	0.32 (0.08)	*F* = 6.22, *p* = 0.01	SZ < HC
*TERT*, mean (SE)	1.49 (<0.01)	1.51 (<0.01)	*F* = 5.03, *p* = 0.03	SZ < HC
*TERC*, mean (SE)	1.52 (<0.01)	1.53 (<0.01)	*F* = 1.04, *p* = 0.31	
*TERT*-to-Telomere Ratio	4.24 (0.06)	4.00 (0.06)	F = 6.68, p = 0.01	SZ > HC
*TERC*-to-Telomere Ratio	4.36 (0.06)	4.11 (0.06)	F = 6.71, p = 0.01	SZ > HC

SZ = schizophrenia spectrum, HC = Healthy Controls, n = number, SD = standard deviation, mg/day = milligrams per day, T/S ratio = telomere to single copy gene ratio. (For telomere length, *TERT* and *TERC*, statistics [F and *p*-value] are based on log transformation and adjusted for age, sex and ethnicity).

### 3.2 Telomere biology and group status

Patients had significantly reduced *TERT* expression compared to healthy controls (*F* = 5.02, *p* = 0.03), but no difference in terms of *TERC* expression (*F* = 1.04, *p* = 0.31). Neither *TERT* nor *TERC* expression was associated with medication use or duration of illness (all *p* > 0.05). However, patients had higher *TERT/TL* (*F* = 6.68, *p* = 0.01) and *TERC*/TL ratio (*F* = 6.71, *p* = 0.01) compared to HC, see Table 1. As already noted in an overlapping sample (Aas et al., 2019) patients had significantly shorter telomeres compared to healthy controls (*F* = 6.22, *p* = 0.01).

### 3.3 Telomere biology and cognitive functioning

No significant associations were observed between any of the telomere biology measures (telomere length, *TERT* or *TERC* expression, *TERT*/TL or *TERC*/TL ratio's) on any of the five cognitive domains (all *p*'s > 0.05) in SZ patients (see Table 2, Table 3, Table 4, Table 5, Table 6) or in healthy controls, HC (see Supplementary Material S3–7).

**Table 2:** Telomere length and cognitive functioning in schizophrenia.

Dependent variable	Std. error	Beta*	Significance	95 % CI	
				Lower	Upper
Working memory	0.13	−0.07	0.22	−0.41	−0.23
Verbal fluency	0.11	−0.78	0.17	−0.38	0.07
Processing speed	0.15	−0.17	0.18	−0.79	−0.18
Verbal memory	0.15	−0.57	0.32	−0.44	0.15
Verbal learning	0.14	−0.05	0.33	−0.41	0.14

Adjusted for age, sex, ethnicity, Mood Stabiliser DDD, Antidepressant DDD, Antipsychotic DDD, Lithium DDD, DDD = Daily Defined Dose.

**Table 3:** *TERT* expression and cognitive functioning in schizophrenia.

Dependent variable	Std. Error	Beta*	Significance	95 % CI	
				Lower	Upper
Working memory	4.20	0.02	0.75	−6.95	9.61
Verbal fluency	3.81	−0.06	0.38	−10.90	4.13
Processing speed	5.18	0.03	0.67	−7.99	12.41
Verbal memory	5.09	0.04	0.52	−6.79	13.29
Verbal learning	4.57	<0.01	0.96	−9.23	8.78

Adjusted for age, sex, ethnicity, Mood Stabiliser DDD, Antidepressant DDD, Antipsychotic DDD, Lithium DDD, DDD = Daily Defined Dose.

**Table 4:** *TERC* expression and cognitive functioning in schizophrenia.

Dependent variable	Std. error	Beta*	Significance	95 % CI	
				Lower	Upper
Working memory	2.78	−0.03	0.67	−4.30	6.66
Verbal fluency	2.53	−0.05	0.41	−7.05	2.91
Processing speed	3.43	−0.06	0.39	−9.75	3.78
Verbal memory	3.37	−0.03	0.66	−8.13	5.16
Verbal learning	3.03	−0.01	0.94	−6.22	5.73

Adjusted for age, sex, ethnicity, Mood Stabiliser DDD, Antidepressant DDD, Antipsychotic DDD, Lithium DDD, DDD = Daily Defined Dose.

**Table 5:** *TERT-*to-telomere ratio and cognitive functioning in schizophrenia.

Dependent variable	Std. error	Beta*	Significance	95 % CI	
				Lower	Upper
Working memory	0.06	0.07	0.34	−0.06	0.18
Verbal fluency	0.06	0.10	0.14	−0.03	0.20
Processing speed	0.08	0.20	0.18	0.08	0.38
Verbal memory	0.07	0.07	0.34	−0.07	0.21
Verbal learning	0.07	0.04	0.59	−0.010	0.17

Adjusted for age, sex, ethnicity, Mood Stabiliser DDD, Antidepressant DDD, Antipsychotic DDD, Lithium DDD, DDD = Daily Defined Dose.

**Table 6:** *TERC-*to-telomere ratio and cognitive functioning in schizophrenia.

Dependent variable	Std. error	Beta*	Significance	95 % CI	
				Lower	Upper
Working memory	0.06	0.07	0.34	−0.06	0.18
Verbal fluency	0.06	0.10	0.15	−0.03	0.19
Processing speed	0.07	0.19	0.18	0.07	0.36
Verbal memory	0.07	0.06	0.38	−0.08	0.20
Verbal learning	0.07	0.03	0.60	−0.10	0.16

Adjusted for age, sex, ethnicity, Mood Stabiliser DDD, Antidepressant DDD, Antipsychotic DDD, Lithium DDD, DDD = Daily Defined Dose.

Sensitive analyses were run for each cognitive wave separately, supporting no significant associations for the telomere biology measurements and cognitive functioning in SZ or in HC (all *p*'s > 0.05), see Supplementary Material S8-S17).

## 4 Discussion

Our study reports reduced *TERT* gene expression, and higher *TERT/TL* and *TERC/TL* ratios in patients with a psychotic disorder compared to healthy individuals. This is in line with reduced telomere length as we have previously published in a larger cohort (Aas et al., 2019), suggesting alteration in telomere biology in patients with a psychotic disorder.

Thus far, only one study has investigated telomerase activity in SZ, indicating a significant reduction in telomerase activity within the patient population compared to unaffected individuals, as well as a reduction in telomerase activity with increased illness severity (Porton et al., 2008). Individuals with particular *TERT* gene variations (rs10069690 & rs2075786) exhibit elevated risk of paranoid SZ, as well as possessing reduced telomere length (Rao et al., 2016).

In line with the current study findings, it could be suggested that it is *TERT* which is the limiting factor in telomere construction, as the levels of *TERC* expression remained unchanged between the clinical and unaffected populations, a theory supported by cancer research (Amin et al., 2023; Liu et al., 2024). Moreover, we found higher *TERT/TL* and *TERC/TL* ratios in SZ patients compared to HC. These ratios serve as a proxy for telomere maintenance efficiency, indicating that although *TERT* is less expressed, the available *TERT* (as well as *TERC*) is being utilised, in order to counteract telomeric attrition. Thus, our study suggests new and novel findings of telomere biology compensatory mechanisms in SZ.

Our findings serve as a dynamic insight into the cellular compensatory mechanisms of SZ patients, in regard to telomere attrition. Such findings can be further contextualized by the fact that *TERT* is responsible for more than direct telomere maintenance, being involved in the cellular stress response (Green et al., 2019), which has been proposed as a significant contributing factor to both telomere degeneration (Lin and Epel, 2022) and SZ manifestation (Pruessner et al., 2017). Our findings of reduced *TERT* expression and higher telomere maintenance and repair processes in SZ, suggest a compensatory mechanism in SZ that may be insufficient in regulating telomere length.

In regard to the association between telomeric factors and cognition, our results indicated no significant associations between telomere length, *TERT* and *TERC* expression and any of the cognitive domains. The lack of a significant findings between telomere biology and cognition contradicts a previous large population study in healthy individuals where longer telomeres were found to be associated with better cognitive performance across domains (Hägg et al., 2017), as well as two studies in SZ (Gurvich et al., 2023 & Czepielewski et al., 2018). However, the study by Gurvich and colleagues was comprised of both schizophrenia spectrum (*N* = 26) and bipolar patients (*N* = 47), compared to HCs (*N* = 113); whilst the study by Czepielewski et al. only assessed verbal memory and had a comparatively small sample (*N* = 46). Nevertheless, both studies did include older participants than those in the current study. Hence, our patients (mean age = 29) may have been too young for telomere length or telomerase expression to influence cognition. To date our study is the first to investigate telomerase expression and cognitive functioning in SZ again suggesting limited role of telomere biology behind cognitive impairments in a relative young group of patients with a psychotic disorder.

### 4.1 Strengths & limitations

The current study is a relatively large, well-characterized sample comprising a total of 758 participants (SZ [*n* = 357] and HCs [*n* = 401]) and is the largest of such studies to date that we are aware of. Moreover, the study provides a comprehensive overview of telomere biology in the clinical and control populations, and its association with cognitive functioning, analyzing not only telomere length, but also providing measures of telomerase expression (*TERT* & *TERC*), and measurements of telomere maintenance efficiency. Nevertheless, there are a few important methodological limitations to consider. First, this study employs a cross-sectional design and thus, we cannot give information on causality. Another methodological limitation is the use of qPCR in measuring telomere length. Whilst qPCR is a validated method often employed in epidemiological research, due to its ability to provide results using only a small amount of DNA (ng; (Lindrose et al., 2021), the qPCR method measures mean telomere length as opposed to proportion or number of short telomeres within a sample (Aas et al., 2019). Therefore, it can be argued that a better estimate of the relationship between telomeres and cognition could have been achieved using a measurement technique with a higher sensitivity to short telomeres. Furthermore, as mentioned, the participants in our study may have been too young (mean age 29), or illness duration too short (median duration 4 years), to find a negative effect of telomere biology (telomere length, telomerase expression and the telomere maintenance and repair ratio) on cognitive functioning.

### 4.2 Conclusion

To conclude, our study contributes to new understanding of telomere biology and its maintenance and repair mechanisms in SZ. Our novel findings include reduced *TERT* expression and higher telomere maintenance and repair processes. However, the role of telomere biology underlying cognitive impairments in SZ seems limited.

## Financial disclosure

This study was funded by a grant from the 10.13039/501100005416Research Council of Norway (#223273) and the 10.13039/501100000265MRC (#MR/W027720/1) and the 10.13039/100010661EU Framework Programme for Research and Innovation H2020 (RealMent; #964874).

## CRediT authorship contribution statement

**Vid Mlakar:** Writing – review & editing, Writing – original draft, Visualization, Validation, Software, Resources, Project administration, Methodology, Investigation, Formal analysis, Data curation, Conceptualization. **Ibrahim Akkouh:** Writing – review & editing, Data curation. **Els F. Halff:** Writing – review & editing, Data curation. **Deepak P. Srivastava:** Data curation, Writing – review & editing. **Viktoria Birkenæs:** Writing – review & editing, Data curation. **Torill Ueland:** Writing – review & editing, Data curation. **Daniel S. Quintana:** Writing – review & editing, Data curation. **Monica B.E.G. Ormerod:** Writing – review & editing, Data curation. **Nils Eiel Steen:** Writing – review & editing, Data curation. **Srdjan Djurovic:** Writing – review & editing, Data curation. **Ole A. Andreassen:** Writing – review & editing, Supervision, Funding acquisition, Data curation. **Monica Aas:** Writing – review & editing, Writing – original draft, Visualization, Validation, Supervision, Software, Resources, Project administration, Methodology, Investigation, Funding acquisition, Formal analysis, Data curation, Conceptualization.

## Declaration of competing interest

Authors declare no conflict of interests.

## Appendix A Supplementary data

Supplementary tables Image 1
