# Supplementary material for: Telomere biology and its maintenance in schizophrenia spectrum disorders: Exploring links to cognition
Source: Schizophr Res. 2024 Oct;272:89–95. doi: 10.1016/j.schres.2024.08.011 (PMC11846775; doi:10.1016/j.schres.2024.08.011)
Supplement: Supplementary file 1 — Supplementary tables [file mmc1.docx]

| *Table S1*. Cognitive domains and their assessment tools with corresponding test battery | | | | | | | | |
| --- | --- | --- | --- | --- | --- | --- | --- | --- |
| Domain | | | Test | | | Neurocognitive Battery | | |
| Working Memory | | | Letter–number sequencing test (WAIS-III)  Letter–number sequencing test (MCCB) | | | 1  2 | | |
| Verbal Fluency | | | Category fluency test (D-KEFS)  Category fluency test (MCCB) | | | 1  2 | | |
| Processing Speed | | | Digit symbol coding test (WAIS-III)  Brief Assessment of Cognition in Schizophrenia | | | 1  2 | | |
| Verbal Memory | | | CVLT, long delay free recall  HVLT, long delay free recall | | | 1  2 | | |
| Verbal Learning | | | CVLT, total number correct over five trials  HVLT, total number correct over three trials | | | 1  2 | | |
| *1 = Simonsen et al., 2010, 2 = MATRICS Consensus Cognitive Battery (MCCB), CVLT = California Verbal Learning Test, HVLT = Hopkins Verbal Learning Test, WAIS-III = Wechsler Adult Intelligence Scale III, D-KEFS = Delis-Kaplan Executive Function System* | | | | | | | | |
| *Table S2.* ANCOVA investigating differences in cognitive functioning between diagnostic groups | | | | | | | | |
| Variable | SZ (n = 357) | HC (n = 401) | | F | df | | Significance | Post Hoc Analysis |
| Working Memory,  mean (SD) | -0.76, (1.16) | 0.00 (1.00) | | 40.04 | 2 | | p < 0.001 | SZ < HC |
| Verbal Fluency,  mean (SD) | -1.27 (1.05) | 0.00 (0.99) | | 108.60 | 2 | | p < 0.001 | SZ < HC |
| Processing Speed,  mean (SD) | -1.32 (1.45) | -0.00 (0.99) | | 99.30 | 2 | | p < 0.001 | SZ < HC |
| Verbal Memory,  mean (SD) | -0.65 (1.36) | -0.00 (1.00) | | 27.05 | 2 | | p < 0.001 | SZ < HC |
| Verbal Learning,  mean (SD) | -0.64 (1.31) | -0.00 (1.00) | | 30.77 | 2 | | p < 0.001 | SZ < HC |
| *SZ = Schizophrenia Spectrum Disorders, HC = Healthy Controls, Covariates = Age, Sex, Ethnicity,* | | | | | | | | |

| *Table S3.* Telomere length and cognitive functioning in healthy controls | | | | | |
| --- | --- | --- | --- | --- | --- |
|  |  |  |  | 95% CI | |
| Dependent Variable | Std. Error | Beta | Significance | Lower | Upper |
| Working Memory | 0.256 | -0.017 | 0.736 | -0.590 | 0.417 |
| Verbal Fluency | 0.255 | -0.011 | 0.828 | -0.557 | 0.446 |
| Processing Speed | 0.248 | -0.035 | 0.481 | -0.662 | 0.312 |
| Verbal Memory | 0.255 | 0.003 | 0.949 | -0.485 | 0.517 |
| Verbal Learning | 0.255 | -0.025 | 0.626 | -0.625 | 0.377 |
| Adjusted for age, sex and ethnicity | | | | | |

| *Table S4.* *TERT* and cognitive functioning in healthy controls | | | | | |
| --- | --- | --- | --- | --- | --- |
|  |  |  |  | 95% CI | |
| Dependent Variable | Std. Error | Beta | Significance | Lower | Upper |
| Working Memory | 0.679 | -0.007 | 0.899 | -1.423 | 1.251 |
| Verbal Fluency | 0.655 | 0.022 | 0.707 | -1.042 | 1.534 |
| Processing Speed | 0.635 | -0.031 | 0.586 | -1.595 | 0.904 |
| Verbal Memory | 0.640 | 0.069 | 0.234 | -0.496 | 2.025 |
| Verbal Learning | 0.663 | 0.048 | 0.412 | -0.760 | 1.849 |
| Adjusted for age, sex and ethnicity | | | | | |

| *Table S5.* *TERC* and cognitive functioning in healthy controls | | | | | |
| --- | --- | --- | --- | --- | --- |
|  |  |  |  | 95% CI | |
| Dependent Variable | Std. Error | Beta | Significance | Lower | Upper |
| Working Memory | 0.590 | -0.034 | 0.568 | -1.498 | 0.824 |
| Verbal Fluency | 0.569 | 0.037 | 0.530 | -0.761 | 1.477 |
| Processing Speed | 0.551 | 0.045 | 0.435 | -0.654 | 1.516 |
| Verbal Memory | 0.557 | 0.035 | 0.550 | -0.764 | 1.431 |
| Verbal Learning | 0.576 | 0.028 | 0.634 | -0.859 | 1.409 |
| Adjusted for age, sex and ethnicity | | | | | |

| *Table S6. TERT*-to-Telomere ratio and cognitive functioning in healthy controls | | | | | |
| --- | --- | --- | --- | --- | --- |
|  |  |  |  | 95% CI | |
| Dependent Variable | Std. Error | Beta | Significance | Lower | Upper |
| Working Memory | 0.075 | 0.012 | 0.841 | -0.132 | 0.162 |
| Verbal Fluency | 0.072 | 0.042 | 0.479 | -0.091 | 0.193 |
| Processing Speed | 0.070 | 0.065 | 0.259 | -0.058 | 0.216 |
| Verbal Memory | 0.071 | 0.013 | 0.828 | -0.124 | 0.154 |
| Verbal Learning | 0.073 | 0.069 | 0.243 | -0.058 | 0.229 |
| Adjusted for chronological age, sex and ethnicity | | | | | |

| *Table S7. TERC-*to-Telomere ratio and cognitive functioning in healthy controls | | | | | |
| --- | --- | --- | --- | --- | --- |
|  |  |  |  | 95% CI | |
| Dependent Variable | Std. Error | Beta | Significance | Lower | Upper |
| Working Memory | 0.072 | 0.009 | 0.878 | -0.130 | 0.152 |
| Verbal Fluency | 0.069 | 0.048 | 0.422 | -0.080 | 0.192 |
| Processing Speed | 0.067 | 0.075 | 0.193 | -0.044 | 0.219 |
| Verbal Memory | 0.068 | 0.013 | 0.831 | -0.119 | 0.148 |
| Verbal Learning | 0.070 | 0.071 | 0.231 | -0.054 | 0.222 |
| Adjusted for chronological age, sex and ethnicity | | | | | |

| *Table S8.* Telomere length and cognitive functioning in schizophrenia spectrum (split batteries) | | | | | | | | | | |
| --- | --- | --- | --- | --- | --- | --- | --- | --- | --- | --- |
| Cognitive Battery 1 | | | | | | Cognitive Battery 2 | | | | |
|  |  |  |  | 95% CI | |  |  |  | 95% CI | |
| Dependent Variable | Std. Error | Beta | Significance | Lower | Upper | Std. Error | Beta | Significance | Lower | Upper |
| Working Memory | 34.403 | 0.114 | 0.130 | -15.533 | 120.251 | 1.107 | -0.127 | 0.129 | -3.878 | 0.499 |
| Verbal Fluency | 2.597 | -0.115 | 0.117 | -9.210 | 1.039 | 2.128 | 0.039 | 0.645 | -3.226 | 5.193 |
| Processing Speed | 4.281 | -0.174 | 0.180 | -19.035 | -2.138 | 3.616 | -0.051 | 0.532 | -9.417 | 4.885 |
| Verbal Memory | 0.925 | -0.161 | 0.028 | -3.877 | -0.226 | 0.983 | 0.083 | 0.374 | -1.072 | 2.827 |
| Verbal Learning | 3.188 | -0.061 | 0.403 | -8.966 | 3.617 | 1.945 | -0.040 | 0.627 | -4.794 | 2.900 |
| Adjusted for chronological age, sex, ethnicity and medication daily defined dose | | | | | | | | | | |

| *Table S9. TERT* expression and cognitive functioning in schizophrenia spectrum (split batteries) | | | | | | | | | | |
| --- | --- | --- | --- | --- | --- | --- | --- | --- | --- | --- |
| Cognitive Battery 1 | | | | | | Cognitive Battery 2 | | | | |
|  |  |  |  | 95% CI | |  |  |  | 95% CI | |
| Dependent Variable | Std. Error | Beta | Significance | Lower | Upper | Std. Error | Beta | Significance | Lower | Upper |
| Working Memory | 114.447 | -0.027 | 0.749 | -262.990 | 189.662 | 4.336 | -0.002 | 0.989 | -8.700 | 8.581 |
| Verbal Fluency | 9.461 | -0.033 | 0.693 | -22.456 | 14.966 | 8.400 | -0.050 | 0.651 | -20.545 | 12.915 |
| Processing Speed | 14.907 | 0.091 | 0.253 | -12.353 | 46.606 | 15.256 | -0.043 | 0.698 | -36.326 | 24.432 |
| Verbal Memory | 3.295 | 0.007 | 0.934 | -6.242 | 6.792 | 4.494 | 0.146 | 0.261 | -3.905 | 14.114 |
| Verbal Learning | 11.316 | 0.030 | 0.714 | -18.223 | 26.535 | 7.458 | -0.019 | 0.863 | -16.141 | 13.561 |
| Adjusted for chronological age, sex, ethnicity and medication daily defined dose | | | | | | | | | | |

| *Table S10. TERC* expression and cognitive functioning in schizophrenia spectrum (split batteries) | | | | | | | | | | |
| --- | --- | --- | --- | --- | --- | --- | --- | --- | --- | --- |
| Cognitive Battery 1 | | | | | | Cognitive Battery 2 | | | | |
|  |  |  |  | 95% CI | |  |  |  | 95% CI | |
| Dependent Variable | Std. Error | Beta | Significance | Lower | Upper | Std. Error | Beta | Significance | Lower | Upper |
| Working Memory | 72.357 | -0.034 | 0.692 | -171.802 | 114.380 | 2.765 | 0.022 | 0.845 | -4.968 | 6.052 |
| Verbal Fluency | 5.978 | -0.050 | 0.548 | -15.427 | 8.222 | 5.373 | -0.046 | 0.670 | -13.003 | 8.399 |
| Processing Speed | 9.460 | -0.047 | 0.560 | -24.241 | 13.176 | 9.812 | -0.034 | 0.749 | -22.690 | 16.385 |
| Verbal Memory | 2.082 | 0.046 | 0.581 | -2.964 | 5.269 | 2.815 | -0.171 | 0.166 | -9.599 | 1.688 |
| Verbal Learning | 7.150 | 0.048 | 0.554 | -9.901 | 18.380 | 4.794 | -0.026 | 0.803 | -10.745 | 8.347 |
| Adjusted for chronological age, sex, ethnicity and medication daily defined dose | | | | | | | | | | |

| *Table S11. TERT*-to-Telomere ratio and cognitive functioning in schizophrenia spectrum (split batteries) | | | | | | | | | | |
| --- | --- | --- | --- | --- | --- | --- | --- | --- | --- | --- |
| Cognitive Battery 1 | | | | | | Cognitive Battery 2 | | | | |
|  |  |  |  | 95% CI | |  |  |  | 95% CI | |
| Dependent Variable | Std. Error | Beta | Significance | Lower | Upper | Std. Error | Beta | Significance | Lower | Upper |
| Working Memory | 7.434 | -0.047 | 0.589 | -18.730 | 10.671 | 0.297 | 0.192 | 0.094 | -0.089 | 1.095 |
| Verbal Fluency | 0.609 | 0.149 | 0.180 | -0.138 | 2.272 | 0.585 | -0.003 | 0.975 | -1.184 | 1.148 |
| Processing Speed | 0.950 | 0.211 | 0.180 | 0.626 | 4.382 | 1.067 | 0.135 | 0.229 | -0.831 | 3.418 |
| Verbal Memory | 0.213 | 0.116 | 0.175 | -0.131 | 0.711 | 0.273 | -0.018 | 0.888 | -0.586 | 0.508 |
| Verbal Learning | 0.736 | 0.016 | 0.849 | -1.315 | 1.595 | 0.526 | 0.045 | 0.681 | -0.830 | 1.263 |
| Adjusted for chronological age, sex, ethnicity and medication daily defined dose | | | | | | | | | | |

| *Table S12. TERC*-to-Telomere ratio and cognitive functioning in schizophrenia spectrum (split batteries) | | | | | | | | | | |
| --- | --- | --- | --- | --- | --- | --- | --- | --- | --- | --- |
| Cognitive Battery 1 | | | | | | Cognitive Battery 2 | | | | |
|  |  |  |  | 95% CI | |  |  |  | 95% CI | |
| Dependent Variable | Std. Error | Beta | Significance | Lower | Upper | Std. Error | Beta | Significance | Lower | Upper |
| Working Memory | 7.214 | -0.048 | 0.581 | -18.253 | 10.280 | 0.290 | 0.192 | 0.095 | -0.087 | 1.068 |
| Verbal Fluency | 0.592 | 0.146 | 0.089 | -0.156 | 2.184 | 0.571 | -0.007 | 0.949 | -1.175 | 1.102 |
| Processing Speed | 0.924 | 0.201 | 0.180 | 0.497 | 4.151 | 1.043 | 0.130 | 0.248 | -0.862 | 3.290 |
| Verbal Memory | 0.206 | 0.121 | 0.158 | -0.115 | 0.701 | 0.268 | -0.046 | 0.724 | -0.633 | 0.442 |
| Verbal Learning | 0.714 | 0.018 | 0.220 | -1.255 | 1.569 | 0.513 | 0.041 | 0.707 | -0.828 | 1.216 |
| Adjusted for chronological age, sex, ethnicity and medication daily defined dose | | | | | | | | | | |

| *Table S13.* Telomere length and cognitive functioning in healthy controls (split batteries) | | | | | | | | | | |
| --- | --- | --- | --- | --- | --- | --- | --- | --- | --- | --- |
| Cognitive Battery 1 | | | | | | Cognitive Battery 2 | | | | |
|  |  |  |  | 95% CI | |  |  |  | 95% CI | |
| Dependent Variable | Std. Error | Beta | Significance | Lower | Upper | Std. Error | Beta | Significance | Lower | Upper |
| Working Memory | 2.209 | -0.095 | 0.642 | -5.597 | 3.520 | 0.750 | -0.010 | 0.842 | -1.623 | 1.325 |
| Verbal Fluency | 9.423 | -0.269 | 0.203 | -31.780 | 7.116 | 1.586 | 0.008 | 0.884 | -2.888 | 3.350 |
| Processing Speed | 9.354 | -0.384 | 0.180 | -39.603 | -0.901 | 2.315 | -0.021 | 0.674 | -5.527 | 3.579 |
| Verbal Memory | 0.076 | -0.274 | 0.119 | -0.280 | 0.034 | 0.467 | 0.008 | 0.882 | -0.849 | 0.988 |
| Verbal Learning | 0.305 | -0.224 | 0.247 | -0.991 | 0.267 | 1.037 | -0.021 | 0.686 | -2.459 | 1.620 |
| Adjusted for chronological age, sex and ethnicity | | | | | | | | | | |

| *Table S14. TERT* expression and cognitive functioning in healthy controls (split batteries) | | | | | | | | | | |
| --- | --- | --- | --- | --- | --- | --- | --- | --- | --- | --- |
| Cognitive Battery 1 | | | | | | Cognitive Battery 2 | | | | |
|  |  |  |  | 95% CI | |  |  |  | 95% CI | |
| Dependent Variable | Std. Error | Beta | Significance | Lower | Upper | Std. Error | Beta | Significance | Lower | Upper |
| Working Memory | 158.771 | 0.427 | 0.719 | -1942.381 | 2092.381 | 1.920 | -0.009 | 0.875 | -4.083 | 3.477 |
| Verbal Fluency | 389.711 | 0.064 | 0.959 | -4926.753 | 4976.753 | 3.949 | 0.022 | 0.709 | -6.299 | 9.246 |
| Processing Speed | 14.434 | -1.000 | 0.180 | -708.389 | -241.602 | 5.767 | -0.029 | 0.619 | -14.219 | 8.484 |
| Verbal Memory | 43.301 | -0.945 | 0.212 | -675.195 | 425.195 | 1.137 | 0.069 | 0.240 | -0.900 | 3.575 |
| Verbal Learning | 404.145 | -0.744 | 0.466 | -5585.152 | 4685.152 | 2.616 | 0.051 | 0.389 | -2.890 | 7.409 |
| Adjusted for chronological age, sex and ethnicity | | | | | | | | | | |

| *Table S15. TERC* expression and cognitive functioning in healthy controls (split batteries) | | | | | | | | | | |
| --- | --- | --- | --- | --- | --- | --- | --- | --- | --- | --- |
| Cognitive Battery 1 | | | | | | Cognitive Battery 2 | | | | |
|  |  |  |  | 95% CI | |  |  |  | 95% CI | |
| Dependent Variable | Std. Error | Beta | Significance | Lower | Upper | Std. Error | Beta | Significance | Lower | Upper |
| Working Memory | 20.349 | 0.621 | 0.573 | -242.441 | 274.682 | 1.681 | -0.042 | 0.475 | -4.511 | 2.107 |
| Verbal Fluency | 28.868 | 0.866 | 0.333 | -316.797 | 416.797 | 3.459 | 0.030 | 0.609 | -5.037 | 8.579 |
| Processing Speed | 70.512 | 0.419 | 0.725 | -863.432 | 928.459 | 5.051 | 0.039 | 0.492 | -6.470 | 13.413 |
| Verbal Memory | 13.724 | 0.712 | 0.495 | -160.444 | 188.313 | 0.998 | 0.032 | 0.584 | -1.417 | 2.512 |
| Verbal Learning | 33.127 | 0.929 | 0.242 | -337.854 | 503.974 | 2.295 | 0.016 | 0.781 | -3.879 | 5.155 |
| Adjusted for chronological age, sex and ethnicity | | | | | | | | | | |

| *Table S16. TERT*-to-Telomere ratio and cognitive functioning in healthy controls (split batteries) | | | | | | | | | | |
| --- | --- | --- | --- | --- | --- | --- | --- | --- | --- | --- |
| Cognitive Battery 1 | | | | | | Cognitive Battery 2 | | | | |
|  |  |  |  | 95% CI | |  |  |  | 95% CI | |
| Dependent Variable | Std. Error | Beta | Significance | Lower | Upper | Std. Error | Beta | Significance | Lower | Upper |
| Working Memory | 2.271 | 0.154 | 0.901 | -28.496 | 29.205 | 0.217 | 0.011 | 0.853 | -0.387 | 0.467 |
| Verbal Fluency | 4.404 | 0.507 | 0.661 | -53.369 | 58.555 | 0.446 | 0.037 | 0.536 | -0.601 | 1.153 |
| Processing Speed | 4.014 | 0.812 | 0.397 | -45.421 | 56.580 | 0.651 | 0.053 | 0.363 | -0.688 | 1.873 |
| Verbal Memory | 0.449 | 0.966 | 0.167 | -4.039 | 7.383 | 0.129 | 0.007 | 0.908 | -0.239 | 0.268 |
| Verbal Learning | 1.070 | 0.991 | 0.086 | -5.759 | 21.445 | 0.295 | 0.052 | 0.385 | -0.325 | 0.839 |
| Adjusted for chronological age, sex, and ethnicity | | | | | | | | | | |

| *Table S17. TERC*-to-Telomere ratio and cognitive functioning in healthy controls (split batteries) | | | | | | | | | | |
| --- | --- | --- | --- | --- | --- | --- | --- | --- | --- | --- |
| Cognitive Battery 1 | | | | | | Cognitive Battery 2 | | | | |
|  |  |  |  | 95% CI | |  |  |  | 95% CI | |
| Dependent Variable | Std. Error | Beta | Significance | Lower | Upper | Std. Error | Beta | Significance | Lower | Upper |
| Working Memory | 1.905 | 0.182 | 0.884 | -13.847 | 24.551 | 0.210 | 0.007 | 0.902 | -0.387 | 0.439 |
| Verbal Fluency | 3.649 | 0.531 | 0.643 | -44.080 | 48.557 | 0.431 | 0.042 | 0.483 | -0.546 | 1.152 |
| Processing Speed | 3.513 | 0.795 | 0.415 | -40.034 | 49.246 | 0.629 | 0.062 | 0.286 | -0.566 | 1.911 |
| Verbal Memory | 0.418 | 0.958 | 0.185 | -3.915 | 6.711 | 0.125 | 0.006 | 0.921 | -0.233 | 0.257 |
| Verbal Learning | 0.717 | 0.994 | 0.069 | -2.474 | 15.740 | 0.286 | 0.051 | 0.390 | -0.317 | 0.809 |
| Adjusted for chronological age, sex, and ethnicity | | | | | | | | | | |
